# (*S*,*S*,*S*,*S*)-Nebivolol hydro­chloride hemihydrate

**DOI:** 10.1107/S1600536812045813

**Published:** 2012-11-14

**Authors:** Yoann Rousselin, Amelie Bruel, Alexandre Clavel

**Affiliations:** a Universite de Bourgogne, ICMUB–UMR6302, 9 avenue Alain Savary, 21000 Dijon, France; b CordenPharma–Synkem, 47 rue de Longvic, 21301, Chenove, France

## Abstract

The asymmetric unit of the title hydrated salt, C_22_H_26_F_2_NO_4_^+^·Cl^−^·0.5H_2_O, consists of an (*S*,*S*,*S*,*S*)-nebivolol {nebivol = bis­[2-(6-fluoro-3,4-dihydro-2*H*-1-benzopyran-2-yl)-2-hy­droxy­eth­yl]ammonium} cation, a chloride anion and a half-occupancy water mol­ecule. The dihedral angle between the mean planes of the benzene rings is 50.34 (12)°. The pyran rings adopt half-chair conformations. The crystal packing features O—H⋯O hydrogen bonds and weak N—H⋯Cl, O—H⋯Cl, and O—H⋯Cl inter­actions, producing layers along (010).

## Related literature

For the synthesis of the enanti­opure title product, see: Jas *et al.* (2011 ▶). For a study of related isomers, see: Cini *et al.* (1990 ▶); Peeters *et al.* (1993 ▶); Tuchalski *et al.* (2006 ▶, 2008 ▶). For pharmacological properties of nebivolol, see: Van Lommen *et al.*, (1990 ▶). For distance computations in water mol­ecules, see: Stewart (2009 ▶). For puckering parameters, see: Cremer & Pople, (1975 ▶).
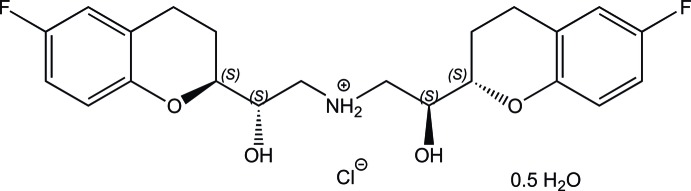


## Experimental

### 

#### Crystal data

C_22_H_26_F_2_NO_4_^+^·Cl^−^·0.5H_2_O
*M* *_r_* = 450.89
Orthorhombic, 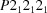

*a* = 7.5173 (3) Å
*b* = 8.1495 (3) Å
*c* = 34.1660 (11) Å
*V* = 2093.09 (13) Å^3^
*Z* = 4
Mo *K*α radiation
μ = 0.23 mm^−1^
*T* = 115 K
0.10 × 0.07 × 0.02 mm

#### Data collection

Nonius Kappa APEXII diffractometer
4782 measured reflections
4782 independent reflections
4271 reflections with *I* > 2σ(*I*)
*R*_int_ = 0.000

#### Refinement

*R*[*F* ^2^ > 2σ(*F* ^2^)] = 0.072
*wR*(*F* ^2^) = 0.137
*S* = 1.27
4782 reflections
290 parameters
3 restraints
H atoms treated by a mixture of independent and constrained refinement
Δρ_max_ = 0.43 e Å^−3^
Δρ_min_ = −0.31 e Å^−3^
Absolute structure: Flack (2003 ▶), 1998 Friedel pairs
Flack parameter: 0.02 (12)

### 

Data collection: *COLLECT* (Nonius, 1998 ▶); cell refinement: *DENZO* (Otwinowski & Minor, 1997 ▶); data reduction: *DENZO*; program(s) used to solve structure: *SIR92* (Altomare *et al.*, 1993 ▶); program(s) used to refine structure: *SHELXL97* (Sheldrick, 2008 ▶); molecular graphics: *ORTEP-3* (Farrugia, 2012 ▶); software used to prepare material for publication: *WinGX* (Farrugia, 2012 ▶).

## Supplementary Material

Crystal structure: contains datablock(s) I, global. DOI: 10.1107/S1600536812045813/jj2154sup1.cif

Structure factors: contains datablock(s) I. DOI: 10.1107/S1600536812045813/jj2154Isup2.hkl

Supplementary material file. DOI: 10.1107/S1600536812045813/jj2154Isup3.cml

Additional supplementary materials:  crystallographic information; 3D view; checkCIF report

## Figures and Tables

**Table 1:** Hydrogen-bond geometry (Å, °)

*D*—H⋯*A*	*D*—H	H⋯*A*	*D*⋯*A*	*D*—H⋯*A*
N1—H1*N*⋯Cl1^i^	0.80 (5)	2.75 (5)	3.333 (4)	131 (4)
N1—H2*N*⋯Cl1^ii^	1.00 (5)	2.20 (5)	3.175 (4)	165 (4)
O2—H2*A*⋯Cl1^iii^	0.84	2.25	3.084 (3)	172
O3—H3⋯O2^iii^	0.84	2.25	2.963 (4)	143
O3—H3⋯O1^iii^	0.84	2.27	2.893 (4)	131
O5—H1*O*⋯O3^iv^	0.94 (2)	2.12 (3)	3.026 (6)	161 (6)
O5—H2*O*⋯Cl1	0.93 (2)	2.28 (3)	3.187 (6)	163 (6)

Symmetry codes: (i) 
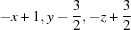
; (ii) 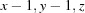; (iii) 
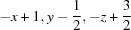
; (iv) 
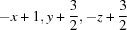
.

**Fig. 1.**
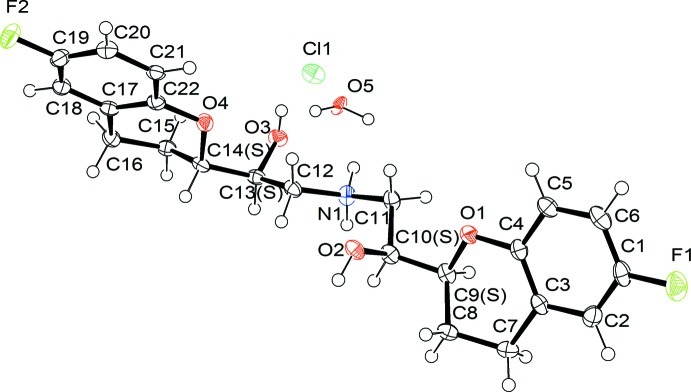
View of the molecular structure of (I) with 50% probability displacement ellipsoids for the non-hydrogen atoms.

**Fig. 2.**
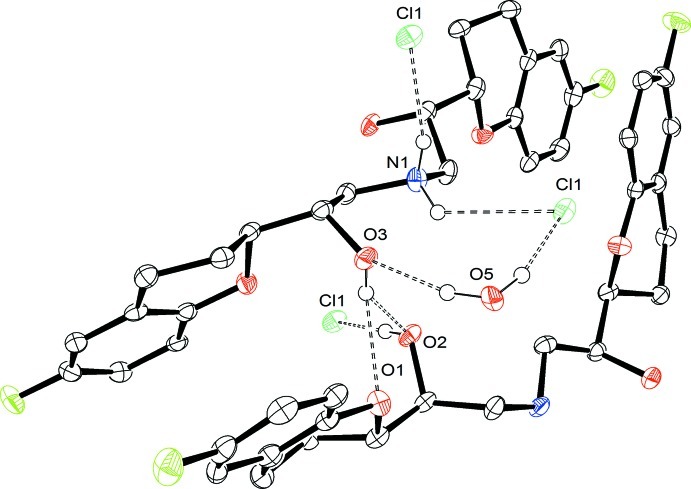
View of the hydrogen-bonding and weak intermolecular interactions in (I). Dashed lines indicate O—H···O hydrogen-bonds and weak N—H···O, N—H···Cl, O—H···Cl, O—H···O and O—H···Cl intermolecular interactions.

**Fig. 3.**
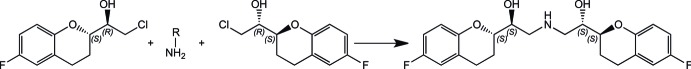
Synthesis of the title compound, (I).

## supplementary crystallographic information

### Comment

(*S*,*S*,*S*,*S*)-Nebivolol is one isomer of the active pharmaceutical ingredient dl-nebivolol which is a highly cardioselective vasodilatory β-receptor blocker used in treatment of hypertension. The chemical structure of nebivolol contains four asymmetric carbon atoms (chiral centers). The combination of all the centers results in 16 theoretical stereoisomers and the total number of isomeric structures is reduced to 10 due to the symmetry plane through the N atom of the molecule. 9 of 10 isomeric structures are known and well described [Tuchalski *et al.* (2006)], here we report the last unknown structure of the title compound, (I), C_22_H_26_F_2_NO_4_^+^Cl^-^.0.5H_2_O, the hydrochloride salt of (*S*,*S*,*S*,*S*)-nebivolol, obtained by total enantio selective synthesis.

The title compound is a salt consisting of a (*S*,*S*,*S*,*S*)-bis[2-(6-fluoro-3,4-dihydro-2H-1-benzopyran-2-yl)-2-hydroxyethyl] ammonium cation, a chloride anion and a water molecule in the asymmetric unit (Fig. 1). The general shape of the cation is strongly influenced by the conformation of the diethylamine chain between the two fluorochroman moieties. The dihedral angle between the mean planes of the two aromatic benzene rings is 50.34 (12)°. Each of the two benzopyran moieties are non-coplanar. The two pyran rings adopt half-chair conformations with total puckering amplitutdes *Q*_T_ of 0.480 (4) (with Θ = 50.5 (5)° and φ = 265.7 (6)°) and 0.489 (4) (with Θ = 129.5 (5)° and φ = 263.4 (6)°), respectively (Cremer & Pople, (1975)). Like other nebivolol isomers, crystal packing in (I) is stabilized by classical O—H···O hydrogen bonds as well as weak N—H···O, N—H···Cl, O—H···Cl, O—H···O and O—H···Cl intermolecular interactions (Fig. 2, Table 1) producing layers along (010).

### Experimental

(*R*)-2-chloro-1-((*S*)-6-fluoro-chroman-2-yl)-1-ethanol was prepared as an enantiopure product in order to obtain the nebivolol isomer.[Jas *et al.* (2011)] A subsequent addition of benzylamine and (*R*)-2-chloro-1-((*S*)-6-fluoro-chroman-2-yl)-1-ethanol was then used to yield the corresponding protected nebivolol. (*S,S,S,S*)-nebivolol hydrochloride was isolated hereafter (Fig. 3).

Preparation of single cristal of (*S*,*S*,*S*,*S*)-nebivolol hydrochloride was performed according to procedure described by Tuchalski *et al.* for (*R,R,R,R*)-nebivolol isomer. The crude product was dissolved at 60 °C in a mixture of ethanol and ethyl acetate (1: 1). The clear solution slowly cooled down to room temperature and the solution left to stand at this temperature. The formation of crystals suitable for X-ray analysis was observed after 8 days. Elemental analysis for (*S,S,S,S*)- Nebivolol hydrochloride + 2 H_2_O, calcd %C 55.29 %H 6.33 %N 2.93, found %C 55.62 %H 6.48 %N 3.52.

### Refinement

The site occupancy factor of the water molecule O5 was refined to close to 0.5. The occupancy was then fixed at 0.5.

The geometric parameters of water molecule were restrained by using *DFIX* restraints. The O—H and H—H distance were restrained to 0.96 (2) Å and 1.50 (2) Å respectively. These distances have been taken from a semi-empirical geometry calculation using MOPAC2009 program (Stewart, 2009) to optimize the molecule with the Austin Model 1 (AM1) approximation

All H atoms, on carbon atoms, were placed at calculated positions using a riding model with C—H = 0.95 Å (aromatic), 0.99 Å (methylene) or 1 Å (methine) with *U*_iso_(H) = 1.2*U*_eq_(C). H atoms on nitrogen atoms and water molecule were located in the Fourier difference maps. Their positional parameters were either refined freely with *U*_iso_(H) = 1.5*U*_eq_(N) or *U*_iso_(H) = 1.5*U*_eq_(O).

TWIN/BASF refinement type was used to determine absolute configuration from anomalous scattering using the Flack method.

### Crystal data

**Table:**

C_22_H_26_F_2_NO_4_^+^·Cl^−^·0.5H_2_O	*F*(000) = 948
*M**_r_* = 450.89	*D*_x_ = 1.431 Mg m^−^^3^
Orthorhombic, *P*2_1_2_1_2_1_	Mo *K*α radiation, λ = 0.71073 Å
Hall symbol: P 2ac 2ab	Cell parameters from 2753 reflections
*a* = 7.5173 (3) Å	θ = 1.0–27.5°
*b* = 8.1495 (3) Å	µ = 0.23 mm^−^^1^
*c* = 34.1660 (11) Å	*T* = 115 K
*V* = 2093.09 (13) Å^3^	Needle, colourless
*Z* = 4	0.10 × 0.07 × 0.02 mm

### Data collection

**Table:**

Nonius Kappa APEXII diffractometer	4271 reflections with *I* > 2σ(*I*)
Radiation source: fine-focus sealed tube	*R*_int_ = 0.000
Horizonally mounted graphite crystal monochromator	θ_max_ = 27.5°, θ_min_ = 2.6°
Detector resolution: 9 pixels mm^-1^	*h* = −9→9
CCD rotation images, thick slices scans	*k* = −10→10
4782 measured reflections	*l* = −43→44
4782 independent reflections	

### Refinement

**Table:**

Refinement on *F*^2^	Secondary atom site location: difference Fourier map
Least-squares matrix: full	Hydrogen site location: inferred from neighbouring sites
*R*[*F*^2^ > 2σ(*F*^2^)] = 0.072	H atoms treated by a mixture of independent and constrained refinement
*wR*(*F*^2^) = 0.137	*w* = 1/[σ^2^(*F*_o_^2^) + (0.*P*)^2^ + 4.746*P*] where *P* = (*F*_o_^2^ + 2*F*_c_^2^)/3
*S* = 1.27	(Δ/σ)_max_ < 0.001
4782 reflections	Δρ_max_ = 0.43 e Å^−^^3^
290 parameters	Δρ_min_ = −0.31 e Å^−^^3^
3 restraints	Absolute structure: Flack (2003), 1998 Friedel pairs
0 constraints	Flack parameter: 0.02 (12)
Primary atom site location: structure-invariant direct methods	

### Special details

**Table:**

Experimental. The X-ray, mass spectrometry and NMR analyzes was recorded in the "Pôle Chimie Moléculaire", the technological platform for chemical analysis and molecular synthesis (http://www.wpcm.fr) which relies on the Institute of the Molecular Chemistry of University of Burgundy and Welience"TM", a Burgundy University private subsidiary.The analytical results concerning identity (NMR and optical rotation) and purity (HPLC and chiral HPLC) are listed below.^1^H and ^13^C NMR measurements were performed in deuterated DMSO on Bruker Avance III, recorded at 500 MHz and 125 MHz, respectively. DMSO-d6 has been used as internal reference. Chemical shifts (δ) and coupling constants are reported respectively in p.p.m. and hertz (Hz).The optical rotation was measured using a UV Visible Perkin Elmer Lambda 12, polarimeter at 589 nm. High-resolution mass spectrometry (HRMS) was performed in ESI a positive mode. The infrared spectrum (IR) was generated by ATR using a Spectrometer Infrared Avatar 370. A scan range of 4000 - 400 cm^-1^ was used.(S,*S*,*S*,*S*)-Nebivolol hydrochloride characterization:δ(^1^H, DMSO-d6, 500 MHz, p.p.m.): 1.77 (2*H*, m); 1.95 (2*H*, m); 2.78 (4*H*, m); 3.21 (4*H*, m); 4.00 (2*H*, m); 4.14 (2*H*, m); 5.79 (2*H*, bs); 6.76 (2*H*, dd); 6.92 (4*H*, m); 8.58 (2*H*, bs).δ(^13^C DMSO-d6, 125.76 MHz, p.p.m.): 22.2; 24.1; 49.5; 67.4; 76.8; 113.6 (23.7); 115.2 (22.5); 117.4 (7.5); 123.7 (7.5); 150.5; 155.9 (235.0).[α]^29^_D_69.6° (c =0.1, THF/water = 4/1)HRMS (ESI) calcd for C_22_H_25_F_2_NO_4_[*M*+H]^+^ m/*z* = 406.18244, found m/*z* = 406.18222.IR (cm^-1^) 3381, 1492, 1215, 812.
Geometry. All e.s.d.'s (except the e.s.d. in the dihedral angle between two l.s. planes) are estimated using the full covariance matrix. The cell e.s.d.'s are taken into account individually in the estimation of e.s.d.'s in distances, angles and torsion angles; correlations between e.s.d.'s in cell parameters are only used when they are defined by crystal symmetry. An approximate (isotropic) treatment of cell e.s.d.'s is used for estimating e.s.d.'s involving l.s. planes.
Refinement. Refinement of *F*^2^ against ALL reflections. The weighted *R*-factor *wR* and goodness of fit *S* are based on *F*^2^, conventional *R*-factors *R* are based on *F*, with *F* set to zero for negative *F*^2^. The threshold expression of *F*^2^ > σ(*F*^2^) is used only for calculating *R*-factors(gt) *etc*. and is not relevant to the choice of reflections for refinement. *R*-factors based on *F*^2^ are statistically about twice as large as those based on *F*, and *R*- factors based on ALL data will be even larger.

### Fractional atomic coordinates and isotropic or equivalent isotropic displacement parameters (Å^2^)

**Table:**

	*x*	*y*	*z*	*U*_iso_*/*U*_eq_	Occ. (<1)
C1	0.4811 (6)	0.0821 (5)	0.97619 (12)	0.0242 (9)	
C2	0.3245 (6)	0.1358 (5)	0.96076 (12)	0.0243 (9)	
H2	0.2368	0.1833	0.9773	0.029*	
C3	0.2926 (5)	0.1210 (5)	0.92044 (12)	0.0211 (8)	
C4	0.4248 (5)	0.0511 (5)	0.89753 (11)	0.0199 (8)	
C5	0.5850 (5)	0.0016 (5)	0.91376 (12)	0.0230 (8)	
H5	0.6748	−0.0436	0.8974	0.028*	
C6	0.6152 (5)	0.0173 (5)	0.95347 (12)	0.0264 (9)	
H6	0.7250	−0.0156	0.9648	0.032*	
C7	0.1216 (5)	0.1825 (5)	0.90272 (12)	0.0237 (8)	
H7A	0.0900	0.2896	0.9145	0.028*	
H7B	0.0245	0.1041	0.9085	0.028*	
C8	0.1409 (6)	0.2016 (5)	0.85853 (11)	0.0209 (8)	
H8A	0.0220	0.2149	0.8465	0.025*	
H8B	0.2119	0.3008	0.8526	0.025*	
C9	0.2313 (5)	0.0525 (5)	0.84167 (11)	0.0212 (8)	
H9	0.1593	−0.0468	0.8483	0.025*	
C10	0.2528 (6)	0.0615 (5)	0.79760 (12)	0.0202 (8)	
H10	0.1331	0.0811	0.7857	0.024*	
C11	0.3266 (6)	−0.0984 (5)	0.78125 (11)	0.0217 (9)	
H11A	0.4582	−0.0942	0.7813	0.026*	
H11B	0.2890	−0.1909	0.7981	0.026*	
C12	0.3360 (5)	−0.0161 (5)	0.70943 (11)	0.0200 (8)	
H12A	0.4675	−0.0160	0.7110	0.024*	
H12B	0.2934	0.0975	0.7135	0.024*	
C13	0.2764 (5)	−0.0781 (5)	0.66940 (12)	0.0195 (8)	
H13	0.1450	−0.0604	0.6676	0.023*	
C14	0.3619 (5)	0.0185 (5)	0.63636 (11)	0.0190 (7)	
H14	0.3190	0.1345	0.6377	0.023*	
C15	0.3219 (5)	−0.0488 (4)	0.59603 (11)	0.0202 (8)	
H15A	0.1915	−0.0528	0.5920	0.024*	
H15B	0.3688	−0.1619	0.5938	0.024*	
C16	0.4070 (5)	0.0597 (5)	0.56468 (12)	0.0219 (9)	
H16A	0.4128	−0.0016	0.5397	0.026*	
H16B	0.3319	0.1580	0.5605	0.026*	
C17	0.5927 (5)	0.1131 (5)	0.57633 (12)	0.0194 (8)	
C18	0.7062 (6)	0.1889 (5)	0.54997 (12)	0.0224 (9)	
H18	0.6683	0.2080	0.5238	0.027*	
C19	0.8729 (6)	0.2363 (5)	0.56154 (12)	0.0253 (9)	
C20	0.9358 (5)	0.2098 (5)	0.59908 (12)	0.0213 (8)	
H20	1.0528	0.2418	0.6063	0.026*	
C21	0.8238 (5)	0.1355 (5)	0.62564 (12)	0.0192 (8)	
H21	0.8637	0.1160	0.6516	0.023*	
C22	0.6531 (5)	0.0890 (4)	0.61476 (11)	0.0192 (8)	
N1	0.2609 (5)	−0.1263 (4)	0.74016 (10)	0.0207 (7)	
H1N	0.279 (6)	−0.221 (6)	0.7347 (13)	0.025*	
H2N	0.130 (6)	−0.109 (5)	0.7406 (13)	0.025*	
O1	0.4083 (3)	0.0325 (3)	0.85757 (8)	0.0220 (6)	
O2	0.3686 (4)	0.1925 (3)	0.78601 (8)	0.0220 (6)	
H2A	0.3087	0.2783	0.7825	0.033*	
O3	0.3073 (4)	−0.2506 (3)	0.66653 (9)	0.0226 (6)	
H3	0.4166	−0.2696	0.6690	0.034*	
O4	0.5511 (3)	0.0167 (4)	0.64353 (7)	0.0209 (6)	
O5	0.8099 (7)	0.8960 (7)	0.82485 (16)	0.0209 (12)*	0.50
H1O	0.760 (10)	0.996 (5)	0.8325 (18)	0.025*	0.50
H2O	0.824 (11)	0.901 (9)	0.7977 (7)	0.025*	0.50
F1	0.5065 (4)	0.0952 (3)	1.01561 (7)	0.0378 (7)	
F2	0.9814 (3)	0.3121 (3)	0.53516 (7)	0.0337 (6)	
Cl1	0.85980 (12)	0.99394 (12)	0.73521 (3)	0.0246 (2)	

### Atomic displacement parameters (Å^2^)

**Table:**

	*U*^11^	*U*^22^	*U*^33^	*U*^12^	*U*^13^	*U*^23^
C1	0.033 (2)	0.021 (2)	0.019 (2)	−0.0051 (18)	−0.0096 (17)	0.0018 (16)
C2	0.036 (2)	0.0172 (19)	0.020 (2)	−0.0008 (17)	0.0037 (18)	−0.0013 (15)
C3	0.025 (2)	0.0158 (18)	0.023 (2)	−0.0002 (16)	−0.0034 (16)	0.0046 (16)
C4	0.024 (2)	0.0164 (18)	0.0198 (19)	−0.0042 (15)	−0.0027 (15)	0.0023 (15)
C5	0.0254 (19)	0.0154 (17)	0.028 (2)	0.0006 (18)	−0.0029 (15)	0.0023 (18)
C6	0.024 (2)	0.024 (2)	0.031 (2)	−0.0009 (19)	−0.0112 (17)	0.0044 (18)
C7	0.018 (2)	0.024 (2)	0.029 (2)	0.0042 (17)	0.0013 (17)	−0.0008 (17)
C8	0.0175 (18)	0.0185 (18)	0.027 (2)	0.0024 (17)	−0.0047 (17)	−0.0017 (16)
C9	0.023 (2)	0.0177 (19)	0.022 (2)	−0.0029 (16)	−0.0046 (17)	0.0019 (16)
C10	0.022 (2)	0.0140 (18)	0.025 (2)	−0.0036 (16)	−0.0039 (16)	0.0000 (16)
C11	0.032 (2)	0.0145 (18)	0.0186 (19)	−0.0021 (17)	−0.0033 (17)	−0.0001 (15)
C12	0.0174 (18)	0.0167 (18)	0.0258 (19)	−0.0008 (17)	−0.0019 (15)	0.0033 (16)
C13	0.0170 (19)	0.0147 (19)	0.027 (2)	0.0007 (15)	0.0007 (16)	−0.0004 (16)
C14	0.0148 (16)	0.0154 (17)	0.0267 (18)	0.0022 (16)	−0.0023 (15)	0.0022 (16)
C15	0.0172 (19)	0.0176 (18)	0.026 (2)	0.0001 (15)	−0.0010 (16)	−0.0018 (16)
C16	0.022 (2)	0.022 (2)	0.0214 (19)	0.0020 (16)	−0.0040 (16)	−0.0003 (16)
C17	0.021 (2)	0.0139 (17)	0.0232 (19)	0.0040 (15)	0.0006 (16)	−0.0033 (15)
C18	0.026 (2)	0.0177 (19)	0.023 (2)	0.0032 (17)	0.0046 (17)	0.0000 (16)
C19	0.024 (2)	0.023 (2)	0.028 (2)	0.0026 (19)	0.0091 (19)	0.0013 (18)
C20	0.0179 (19)	0.0192 (19)	0.027 (2)	−0.0015 (16)	−0.0005 (16)	−0.0034 (17)
C21	0.0170 (19)	0.0158 (18)	0.025 (2)	0.0044 (15)	0.0020 (16)	−0.0004 (15)
C22	0.0209 (19)	0.0123 (17)	0.0243 (19)	0.0022 (16)	0.0019 (17)	−0.0018 (15)
N1	0.0265 (18)	0.0151 (16)	0.0207 (18)	−0.0058 (15)	−0.0025 (15)	−0.0021 (14)
O1	0.0213 (14)	0.0252 (15)	0.0193 (13)	0.0044 (12)	−0.0030 (11)	0.0007 (12)
O2	0.0263 (15)	0.0118 (12)	0.0280 (15)	−0.0005 (12)	−0.0028 (13)	0.0022 (11)
O3	0.0255 (15)	0.0145 (13)	0.0279 (15)	0.0006 (11)	0.0013 (13)	−0.0016 (12)
O4	0.0178 (13)	0.0246 (14)	0.0204 (13)	−0.0001 (12)	−0.0017 (10)	0.0044 (12)
F1	0.0514 (18)	0.0392 (15)	0.0229 (13)	0.0007 (14)	−0.0093 (13)	−0.0011 (12)
F2	0.0313 (14)	0.0413 (15)	0.0285 (14)	−0.0062 (12)	0.0108 (12)	0.0057 (12)
Cl1	0.0251 (4)	0.0197 (4)	0.0289 (5)	0.0010 (4)	−0.0055 (4)	−0.0040 (4)

### Geometric parameters (Å, º)

**Table:**

C1—C2	1.362 (6)	C12—H12B	0.9900
C1—F1	1.365 (5)	C13—O3	1.429 (5)
C1—C6	1.377 (6)	C13—C14	1.519 (5)
C2—C3	1.403 (6)	C13—H13	1.0000
C2—H2	0.9500	C14—O4	1.443 (4)
C3—C4	1.387 (6)	C14—C15	1.513 (5)
C3—C7	1.507 (6)	C14—H14	1.0000
C4—O1	1.379 (5)	C15—C16	1.529 (5)
C4—C5	1.386 (5)	C15—H15A	0.9900
C5—C6	1.382 (5)	C15—H15B	0.9900
C5—H5	0.9500	C16—C17	1.516 (5)
C6—H6	0.9500	C16—H16A	0.9900
C7—C8	1.525 (5)	C16—H16B	0.9900
C7—H7A	0.9900	C17—C18	1.386 (6)
C7—H7B	0.9900	C17—C22	1.403 (5)
C8—C9	1.506 (5)	C18—C19	1.370 (6)
C8—H8A	0.9900	C18—H18	0.9500
C8—H8B	0.9900	C19—F2	1.364 (5)
C9—O1	1.446 (5)	C19—C20	1.384 (6)
C9—C10	1.516 (5)	C20—C21	1.378 (6)
C9—H9	1.0000	C20—H20	0.9500
C10—O2	1.433 (5)	C21—C22	1.389 (5)
C10—C11	1.523 (5)	C21—H21	0.9500
C10—H10	1.0000	C22—O4	1.379 (5)
C11—N1	1.505 (5)	N1—H1N	0.80 (5)
C11—H11A	0.9900	N1—H2N	1.00 (5)
C11—H11B	0.9900	O2—H2A	0.8400
C12—N1	1.492 (5)	O3—H3	0.8400
C12—C13	1.525 (5)	O5—H1O	0.94 (2)
C12—H12A	0.9900	O5—H2O	0.93 (2)
			
C2—C1—F1	118.5 (4)	O3—C13—C14	113.0 (3)
C2—C1—C6	122.5 (4)	O3—C13—C12	109.9 (3)
F1—C1—C6	118.9 (4)	C14—C13—C12	111.8 (3)
C1—C2—C3	120.0 (4)	O3—C13—H13	107.3
C1—C2—H2	120.0	C14—C13—H13	107.3
C3—C2—H2	120.0	C12—C13—H13	107.3
C4—C3—C2	117.9 (4)	O4—C14—C15	110.3 (3)
C4—C3—C7	121.4 (4)	O4—C14—C13	106.6 (3)
C2—C3—C7	120.7 (4)	C15—C14—C13	113.9 (3)
O1—C4—C5	116.2 (4)	O4—C14—H14	108.6
O1—C4—C3	122.6 (4)	C15—C14—H14	108.6
C5—C4—C3	121.1 (4)	C13—C14—H14	108.6
C6—C5—C4	120.6 (4)	C14—C15—C16	110.2 (3)
C6—C5—H5	119.7	C14—C15—H15A	109.6
C4—C5—H5	119.7	C16—C15—H15A	109.6
C1—C6—C5	117.9 (4)	C14—C15—H15B	109.6
C1—C6—H6	121.0	C16—C15—H15B	109.6
C5—C6—H6	121.0	H15A—C15—H15B	108.1
C3—C7—C8	110.5 (3)	C17—C16—C15	111.5 (3)
C3—C7—H7A	109.5	C17—C16—H16A	109.3
C8—C7—H7A	109.5	C15—C16—H16A	109.3
C3—C7—H7B	109.5	C17—C16—H16B	109.3
C8—C7—H7B	109.5	C15—C16—H16B	109.3
H7A—C7—H7B	108.1	H16A—C16—H16B	108.0
C9—C8—C7	109.8 (3)	C18—C17—C22	118.1 (4)
C9—C8—H8A	109.7	C18—C17—C16	121.6 (4)
C7—C8—H8A	109.7	C22—C17—C16	120.2 (4)
C9—C8—H8B	109.7	C19—C18—C17	120.1 (4)
C7—C8—H8B	109.7	C19—C18—H18	120.0
H8A—C8—H8B	108.2	C17—C18—H18	120.0
O1—C9—C8	111.3 (3)	F2—C19—C18	119.0 (4)
O1—C9—C10	106.3 (3)	F2—C19—C20	118.6 (4)
C8—C9—C10	112.9 (3)	C18—C19—C20	122.4 (4)
O1—C9—H9	108.8	C21—C20—C19	118.0 (4)
C8—C9—H9	108.8	C21—C20—H20	121.0
C10—C9—H9	108.8	C19—C20—H20	121.0
O2—C10—C9	112.0 (3)	C20—C21—C22	120.6 (4)
O2—C10—C11	108.3 (3)	C20—C21—H21	119.7
C9—C10—C11	111.2 (3)	C22—C21—H21	119.7
O2—C10—H10	108.4	O4—C22—C21	116.1 (3)
C9—C10—H10	108.4	O4—C22—C17	123.2 (4)
C11—C10—H10	108.4	C21—C22—C17	120.7 (4)
N1—C11—C10	110.6 (3)	C12—N1—C11	116.2 (3)
N1—C11—H11A	109.5	C12—N1—H1N	110 (3)
C10—C11—H11A	109.5	C11—N1—H1N	108 (3)
N1—C11—H11B	109.5	C12—N1—H2N	107 (3)
C10—C11—H11B	109.5	C11—N1—H2N	107 (3)
H11A—C11—H11B	108.1	H1N—N1—H2N	108 (4)
N1—C12—C13	108.7 (3)	C4—O1—C9	116.2 (3)
N1—C12—H12A	109.9	C10—O2—H2A	109.5
C13—C12—H12A	109.9	C13—O3—H3	109.5
N1—C12—H12B	109.9	C22—O4—C14	115.0 (3)
C13—C12—H12B	109.9	H1O—O5—H2O	107 (3)
H12A—C12—H12B	108.3		
			
F1—C1—C2—C3	−178.7 (4)	C12—C13—C14—C15	174.2 (3)
C6—C1—C2—C3	2.2 (6)	O4—C14—C15—C16	−62.4 (4)
C1—C2—C3—C4	−0.1 (6)	C13—C14—C15—C16	177.8 (3)
C1—C2—C3—C7	−178.9 (4)	C14—C15—C16—C17	41.5 (4)
C2—C3—C4—O1	−179.2 (4)	C15—C16—C17—C18	169.2 (4)
C7—C3—C4—O1	−0.4 (6)	C15—C16—C17—C22	−11.9 (5)
C2—C3—C4—C5	−1.7 (6)	C22—C17—C18—C19	0.7 (6)
C7—C3—C4—C5	177.1 (4)	C16—C17—C18—C19	179.7 (4)
O1—C4—C5—C6	179.1 (4)	C17—C18—C19—F2	−179.3 (4)
C3—C4—C5—C6	1.5 (6)	C17—C18—C19—C20	0.8 (6)
C2—C1—C6—C5	−2.4 (6)	F2—C19—C20—C21	178.9 (3)
F1—C1—C6—C5	178.5 (4)	C18—C19—C20—C21	−1.2 (6)
C4—C5—C6—C1	0.5 (6)	C19—C20—C21—C22	0.1 (6)
C4—C3—C7—C8	−16.2 (5)	C20—C21—C22—O4	−179.6 (3)
C2—C3—C7—C8	162.5 (4)	C20—C21—C22—C17	1.5 (6)
C3—C7—C8—C9	45.4 (5)	C18—C17—C22—O4	179.2 (3)
C7—C8—C9—O1	−61.5 (4)	C16—C17—C22—O4	0.3 (6)
C7—C8—C9—C10	179.1 (3)	C18—C17—C22—C21	−1.8 (6)
O1—C9—C10—O2	−57.6 (4)	C16—C17—C22—C21	179.2 (3)
C8—C9—C10—O2	64.6 (4)	C13—C12—N1—C11	171.7 (3)
O1—C9—C10—C11	63.8 (4)	C10—C11—N1—C12	70.9 (4)
C8—C9—C10—C11	−174.0 (3)	C5—C4—O1—C9	168.0 (3)
O2—C10—C11—N1	−86.3 (4)	C3—C4—O1—C9	−14.4 (5)
C9—C10—C11—N1	150.1 (3)	C8—C9—O1—C4	45.4 (4)
N1—C12—C13—O3	−48.4 (4)	C10—C9—O1—C4	168.7 (3)
N1—C12—C13—C14	−174.6 (3)	C21—C22—O4—C14	160.5 (3)
O3—C13—C14—O4	−72.2 (4)	C17—C22—O4—C14	−20.5 (5)
C12—C13—C14—O4	52.3 (4)	C15—C14—O4—C22	51.4 (4)
O3—C13—C14—C15	49.7 (4)	C13—C14—O4—C22	175.5 (3)

### Hydrogen-bond geometry (Å, º)

**Table:**

*D*—H···*A*	*D*—H	H···*A*	*D*···*A*	*D*—H···*A*
N1—H1*N*···Cl1^i^	0.80 (5)	2.75 (5)	3.333 (4)	131 (4)
N1—H2*N*···Cl1^ii^	1.00 (5)	2.20 (5)	3.175 (4)	165 (4)
O2—H2*A*···Cl1^iii^	0.84	2.25	3.084 (3)	172
O3—H3···O2^iii^	0.84	2.25	2.963 (4)	143
O3—H3···O1^iii^	0.84	2.27	2.893 (4)	131
O5—H1*O*···O3^iv^	0.94 (2)	2.12 (3)	3.026 (6)	161 (6)
O5—H2*O*···Cl1	0.93 (2)	2.28 (3)	3.187 (6)	163 (6)

Symmetry codes: (i) −*x*+1, *y*−3/2, −*z*+3/2; (ii) *x*−1, *y*−1, *z*; (iii) −*x*+1, *y*−1/2, −*z*+3/2; (iv) −*x*+1, *y*+3/2, −*z*+3/2.
